# Role of cerebellum in sleep-dependent memory processes

**DOI:** 10.3389/fnsys.2023.1154489

**Published:** 2023-04-18

**Authors:** Andrew Jackson, Wei Xu

**Affiliations:** ^1^Institute of Biosciences, Newcastle University, Newcastle upon Tyne, United Kingdom; ^2^Centre for Discovery Brain Sciences, The University of Edinburgh, Edinburgh, United Kingdom

**Keywords:** cerebellum, motor cortex, sleep, learning, motor control

## Abstract

The activities and role of the cerebellum in sleep have, until recently, been largely ignored by both the sleep and cerebellum fields. Human sleep studies often neglect the cerebellum because it is at a position in the skull that is inaccessible to EEG electrodes. Animal neurophysiology sleep studies have focussed mainly on the neocortex, thalamus and the hippocampus. However, recent neurophysiological studies have shown that not only does the cerebellum participate in the sleep cycle, but it may also be implicated in off-line memory consolidation. Here we review the literature on cerebellar activity during sleep and the role it plays in off-line motor learning, and introduce a hypothesis whereby the cerebellum continues to compute internal models during sleep that train the neocortex.

## Introduction

While sleep plays important roles in metabolic regulation, energy conservation and cellular restoration, it has been described as an activity “of the brain, by the brain and for the brain” (Hobson, 2005), taking up one third of an adult human’s daily life. Despite the fact that the cerebellum contains over 80% of the brain’s neurons (Herculano-Houzel, 2009), this brain structure has until recently been an “uncharted land in sleep research” (Canto et al., 2017). This is likely due to the importance of EEG recordings in sleep studies, and the difficulty of obtaining EEG recordings from the cerebellum as it is overlain with neck muscles. Additionally cerebellar local neuronal circuitry and a highly folded cortical morphology may preclude it from producing sufficiently synchronous electrical activity, giving the impression that the cerebellum produces very little scalp EEG signal (Andersen et al., 2020). Happily, this situation is starting to change. Recent neurophysiological studies have shown that the cerebellum actively participates in sleep and may in fact shape sleep architecture and contribute to off-line memory consolidation (Xu et al., 2021; Torres-Herraez et al., 2022). Perhaps the main reason for the hitherto neglected status of the cerebellum in sleep is the overshadowing nature of its prominent and well-studied role in coordinating movements (Holmes, 1939; Levine, 2007) by representing and updating internal models of the body and environment (Wolpert et al., 1998). That no voluntary movement take place during sleep may also have led cerebellar researchers away from studying this brain state.

During waking movements the cerebellum is thought to compute an internal model of the motor apparatus by providing predictive feedback to on-going motor output (Wolpert et al., 1998). The provision of movement outcome predictions, combined with on-going sensory feedback, enables timely adjustments of neocortical motor outputs on a moment-by-moment basis. We propose a theoretical framework whereby the cerebellum continues to compute internal models in sleep to provide a simulated environment that supports off-line motor learning. The purpose of this review is to collate prior results of cerebellar sleep activity and evidence of cerebellum-dependent learning during sleep into such a theoretical framework.

## Sleep and declarative learning

The sleep cycle is segmented into rapid eye movement (REM) and non-REM phases, with the latter conventionally divided into three stages. The EEG activity from the neocortex (largely used to define different sleep stages) shows a progressive increase of neuronal synchrony as the brain falls deeper into sleep from stage 1 to 3. Stage 1 (light sleep) occurs at the beginning of sleep and only forms around 5% of total sleep duration. It is characterised by low amplitude mixed frequency EEG signals. Deepening sleep progresses to stage 2, comprising 45% of total sleep time and is defined by the presence of sleep spindles and/or K complexes (Colrain, 2005; Fernandez and Luthi, 2020). Sleep spindles are waxing and waning oscillations in the 9–16 Hz range (Fernandez and Luthi, 2020), and have been implicated in off-line consolidation of both procedural and semantic memories (Fogel and Smith, 2006, 2011; Nishida and Walker, 2007; Tamaki et al., 2008; Mölle et al., 2011; Fogel et al., 2017; Latchoumane et al., 2017; Boutin and Doyon, 2020). In addition to enhancing neocortical plasticity (Niethard et al., 2018) they are also thought to play a role in gating out afferent sensory transmission to the neocortex (Wimmer et al., 2012). Stage 3 sleep (deepest sleep, also known as slow-wave sleep) occupies 25% of total sleep duration and is characterised by high amplitude low frequency EEG signals mostly in the delta (1–4 Hz) and low (<1 Hz) range. Finally, REM sleep produces awake-like EEG signals but with concomitant muscle atonia. This stage is when most dreams occur and is not a restful period for the brain. It occupies 25% of sleep time and increases in frequency and duration with time spent asleep. During healthy sleep, for each sleep cycle the brain progresses through these stages sleep and this cycle repeats multiple times per sleep period, each of which in humans take approximately 100 min. For a precise and comprehensive definition of each sleep stage, see “The AASM Manual for the Scoring of Sleep and Associated Events” (Berry et al., 2015).

Much of the neuroscience of sleep has been concerned with sleep-dependent consolidation of declarative memories (Yaroush et al., 1971; Fowler et al., 1973; Plihal and Born, 1997), thought to occur via reactivations of memory-related neuronal patterns in the hippocampus that are transferred to the neocortex for long-term storage. Such a mechanism is supported by the observed increase in neocortical slow waves and sleep spindles in human EEG after declarative learning (Gais and Born, 2004). Additionally, numerous animal studies suggest that hippocampal sleep replay of neuronal patterns associated with waking episodic experiences is important for encoding them into memory (Pfeiffer, 2020). These replayed neuronal patterns then undergo consolidation where the memory is stabilised in the neocortex via hippocampal-neocortical interactions (Siapas and Wilson, 1998; Sirota et al., 2003; Wiltgen et al., 2004; Wierzynski et al., 2009). This stabilisation is thought to involve spontaneous reactivation of experience-related neuronal patterns during sleep (Wilson and McNaughton, 1994; Lee and Wilson, 2002; Ji and Wilson, 2007) that are phase-locked to sharp wave ripples in a temporally compressed manner (Diba and Buzsaki, 2007; Buzsaki, 2015; Joo and Frank, 2018), which are in turn phase-locked to sleep spindles (Clemens et al., 2007).

## Sleep and procedural learning

The cerebellum has long been implicated in the acquisition of procedural skills (De Zeeuw and Ten Brinke, 2015), for example adaptation of the vestibulo-ocular reflex (Lisberger, 1988; Lisberger, 1998), eye-blink conditioning (Boele et al., 2010) and visuomotor adaptation during reaching movements, wherein both simple and complex spikes are thought to encode sensory prediction errors (Kitazawa et al., 1998; Streng et al., 2018; Tzvi et al., 2022). The cerebellum has also been linked to sequence learning [such as pressing a set of keys in a particular order (Buijink et al., 2015; Doyon et al., 2002; Hikosaka et al., 1998)] where cerebellar nuclei have been shown to play a role in storing learned sequences. Despite this focus on sleep and declarative memory consolidation, there is also strong evidence that these cerebellar-dependent procedural memories also undergo sleep dependent changes.

**FIGURE 1:**
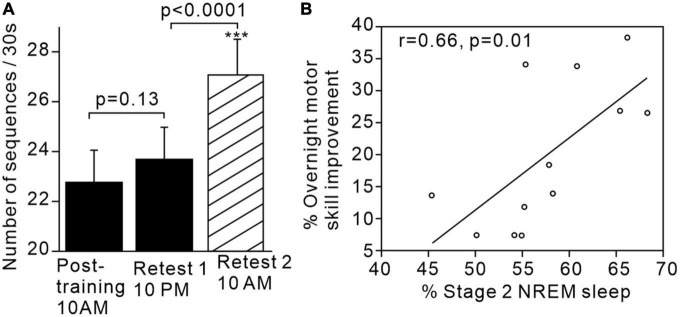
Motor skills improve after sleep and are associated with increased proportion of stage 2 sleep. **(A)** Significant increase in motor sequence performance after sleep but not after the same time period awake. **(B)** Positive correlation between proportion of stage 2 sleep and overnight motor performance improvement. Figures reproduced with permission from Walker et al. (2002). ***p < 0.002.

For example, Walker et al. (2002) demonstrated that subjects’ performance on a learned sequential finger tapping task showed significantly greater improvement after sleep compared to after the same period of time spent awake (Figures 1A, B). Additionally, performance improvement was significantly and positively correlated with the proportion of stage 2 sleep in the intervening sleep period between learning and retest. The same research group postulated that sleep-dependent offline learning of motor skills takes place via an independent process to online practice learning (Walker et al., 2003).

Imaging studies also combining fMRI with motor sequence, visuomotor and timing tasks also suggest a link between cerebellum-dependent procedural learning and sleep. For example, the dentate nucleus of the cerebellum shows increased functional connectivity with the superior temporal sulcus after sleep while learning a pursuit task, but this is reduced after sleep deprivation (Maquet et al., 2003). Increased cerebellar activity after sleep (compared to an equivalent time awake) correlates with improved performance on a finger sequence learning task (Walker et al., 2005). Moreover, brain state (sleep vs. wake) during the retention period of a motor timing task modulates subsequent cerebellar activity during task performance (Lewis et al., 2011). Thus, sleep-dependent processes seem to play an important role in driving the changed patterns of activity across both the neocortex and cerebellum associated with repeated sessions of procedural learning over multiple days (Doyon et al., 2002).

## Cerebellar activity during sleep

Invasive animal neurophysiological studies have focussed mainly on the motor-control functions of the cerebellum (Manto et al., 2012) while studies investigating cerebellar activity during sleep have been relatively sparse, non-cohesive and somewhat lacking in clear hypotheses. Nevertheless, a review of this literature with the benefit of hindsight offers up some early clues of a cerebellar role in sleep. For example, it was clear from pioneering animal studies that electrical stimulation of the cerebellum could either put an animal to sleep or wake it up (Simkina, 1932; Clark and Ward, 1952; Roelofs et al., 1963), and as early as 1961 it was known that stimulation of the cerebellar pyramis and uvula could induce sleep spindles in neocortical EEG (Sawyer et al., 1961). Animal lesion studies have shown sleep to be affected post cerebellar lesion, with the most common finding being increased drowsiness during waking, decreased slow wave sleep and increased REM sleep (De Andres and Reinoso-Suarez, 1979; Cunchillos and De Andres, 1982; Paz et al., 1982). Human clinical studies have largely corroborated these findings, with sleep reported to be more fragmented both in congenital cerebellar disorders and after cerebellar injury, again with concomitant daytime drowsiness, poorer sleep and REM sleep abnormalities being prevalent (Freedman and Rourke, 1954; Bergonzi et al., 1981; Pedroso et al., 2017). Additionally, it is interesting to note that early cerebellar damage or cerebellar abnormalities are associated with autism, attention deficit-hyperactivity disorder (ADHD), dyslexia (Becker and Stoodley, 2013; Stoodley and Stein, 2013; Stoodley, 2014, 2016; Wang et al., 2014) and schizophrenia (Andreasen and Pierson, 2008), and all of these conditions are also associated with disturbed sleep (Devnani and Hegde, 2015; Carotenuto et al., 2016; Smith and Henderson, 2016; Kaskie et al., 2017).

With hindsight, these findings collectively should have produced compelling evidence already by the late 1970s that the cerebellum may play an important role in shaping sleep architecture and the sleep-wake cycle (Zhang et al., 2020). However, this did little to shift the centre of gravity of sleep research away from the neocortex and thalamus (Steriade, 2003). For example, a seminal study by Steriade et al. (1987) demonstrating the disappearance of neocortical sleep spindles after thalamic lesion did not consider the possibility that other brain regions projecting to the thalamus (e.g., the cerebellum) might also contribute to neocortical spindles via the thalamus. Where the sleep neurophysiology of the cerebellum was studied, it was found to be broadly comparable to the neocortex in that cerebellar Purkinje cell simple spikes had the highest firing rate in during REM sleep (Mano, 1970; Marchesi and Strata, 1971; Hobson and McCarley, 1972). However there were disagreements about complex spike rates in different stages of sleep. For example Marchesi and Strata reported complex spikes as having higher firing rates during REM sleep compared to non-REM sleep (Marchesi and Strata, 1970, 1971) whereas Mano described the opposite (Mano, 1970). Nevertheless the general consensus was that, as far as simples spikes were concerned, Purkinje cells were modulated similarly to neocortical neurons during the sleep cycle [possibly in part be due to their reduced responsiveness to glutamate during slow wave sleep (Andre and Arrighi, 2001)]. Despite these early findings, and the extensive and bidirectional traffic between the cerebellum and neocortex (Bostan et al., 2013), very few studies have subsequently investigated the connectivity between cerebellum and cerebrum during sleep.

**FIGURE 2:**
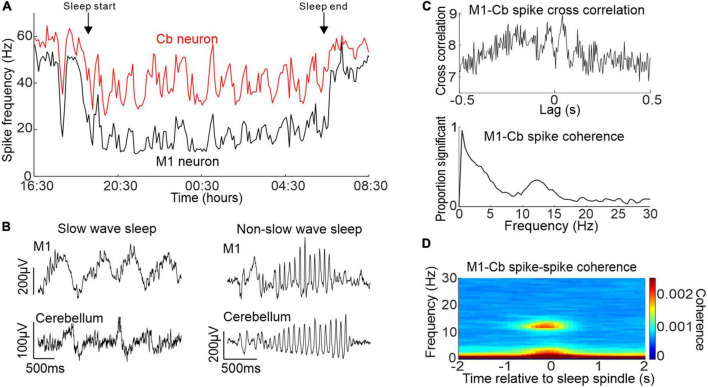
Activity of M1 and cerebellum during sleep. **(A)** Simultaneously recorded M1 and cerebellar neuronal spiking rate over an entire sleep period. **(B)** Example simultaneous M1 and cerebellar slow oscillations (left) and sleep spindles (right). **(C)** Top: example cross-correlation between an M1 and cerebellar spike train during sleep showing both low frequency and spindle frequency components. Bottom: proportion of significant spike-to-spike coherence between pairs of M1 and cerebellar neurons in sleep. **(D)** M1-cerebellar spike coherence aligned by neocortical sleep spindles. Figures adapted with permission from Xu et al. (2021).

To address this gap in knowledge, we carried out a study in monkeys which showed that primary motor cortex (M1) and cerebellar activity during sleep exhibited correlations at several frequency bands (Xu et al., 2021). Using a wearable recording device during natural sleep we confirmed that M1 and cerebellar spike firing rates showed similar changes in activity that were modulated by the sleep cycle (Figure 2A). Moreover, we found that both brain regions showed slow oscillations and sleep spindles that were correlated with each other (Figure 2B). This was accompanied by increased coherence between M1 and cerebellar spiking activity at these same frequency bands (Figure 2C) occurring during identified neocortical sleep spindles (Figure 2D). To our knowledge, this was the first report of sleep spindle oscillations in the cerebellum, although a previous imaging study had found increased cerebellar BOLD signals that were associated with sleep spindles (Schabus et al., 2007). Since sleep spindles have been implicated in procedural memory consolidation [e.g, (Fogel and Smith, 2006)], this raises the intriguing possibility of a role for the cerebellum in this process.

## Cerebellar-neocortical interactions during sleep spindles

**FIGURE 3:**
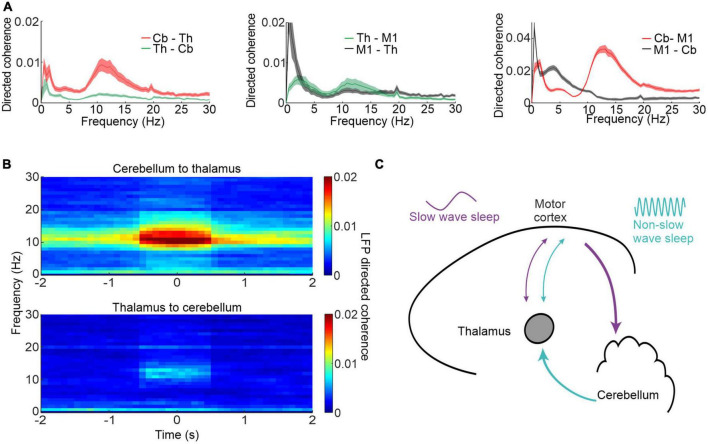
Cerebellar-M1 interactions during sleep spindles. **(A)** Directed coherence between thalamus (Th) and cerebellum (Cb, left), thalamus and M1 (centre) and cerebellum and M1 (right). **(B)** Directed coherence between cerebellum and thalamus aligned by neocortical spindles. **(C)** Schematic showing neocortex-cerebellar interactions during different phases of sleep and at different frequencies. Figures adapted with permission from Xu et al. (2021).

Our study also revealed a surprising directionality of causal influence between the cerebellum, thalamus and neocortex [Figure 3A; Canto et al., 2017)]. Using spectral Granger’s causality analysis (Kamiński et al., 2001) of simultaneously recorded local field potentials (LFPs), we found directed coherence at spindle frequencies was greatest from the cerebellum to the thalamus and M1. Importantly this spindle-band directed coherence was largest during identified neocortical spindles (Figure 3B) raising the possibility that the cerebellum contributes, via pathways through the thalamus, to sleep spindles. Figure 3C show a schematic summary of the key findings, whereby there is a causal influence mainly in the cerebellum-to-M1 direction during sleep spindles as opposed to a causal influence in the reverse direction at low frequencies during slow wave sleep.

These results place the cerebellum in an important position in the sleep process and complicate the decades-old sleep-research doctrine that neocortical sleep spindles arise solely from the thalamus (Steriade et al., 1985, 1987). Moreover, the spindle drive from the cerebellum implies that it may even be involved in shaping sleep architecture, since sleep spindles reduce environmental influences on neocortical activity and thereby facilitate deeper sleep (Steriade et al., 1993; Buzsaki and Draguhn, 2004). Such a hypothesis could explain why cerebellar lesions decrease in the amount of slow wave sleep.

How might the cerebellum generate oscillations in the sleep spindle frequency range (9–16 Hz) The spindle-generating mechanism is fairly well-described in the thalamus, via reciprocal interactions between thalamocortical and thalamic reticular neurons. Activation of the GABAergic thalamic reticular neurons activates GABAa and GABAb receptors on thalamocortical neurons, leading to hyperpolarisations which then causes a rebound excitation. Conversely, rebound excitation in thalamocortical neurons can then cause excitation and spike bursting in thalamic reticular neurons, thereby creating reverberating activity in the loop at spindle frequencies [for detailed review see McCormick and Bal (1997)].

Are there similar circuits in the cerebellum that could plausibly generate spindle-band oscillations? There certainly exists reverberating loops within the cerebellar cortex and between the cerebellar cortex and its related nuclei. For example, cerebellar Purkinje cells inhibit neurons of the deep cerebellar nuclei which then show rebound excitation (Aizenman and Linden, 1999). Inhibitory cells from the deep cerebellar nuclei then project to the inferior olive to influence its intrinsic rhythmicity by altering the strength of its gap junctions (Andersson et al., 1988; Zeeuw et al., 1988, 1997; Fredette and Mugnaini, 1991; Teune et al., 1998; Svensson et al., 2006). The inferior olive then projects powerful excitatory inputs back to cerebellar Purkinje cells in the form of complex spikes (Eccles et al., 1966). This cerebellar olivary system is thought to be responsible for generating 1–9 Hz oscillations in the cerebellum (Lang et al., 2006; Van Der Giessen et al., 2008). Another excitation-inhibition loop in the cerebellum takes the form of granule cell—Golgi cell interconnections (Palay and Chan-Palay, 1974). Granule cells and Golgi cells both receive excitatory mossy fibre inputs. Granule cells then go on to excite Golgi cells (via its parallel fibres) which then sends feedback inhibition to granule cell dendrites (D’Angelo et al., 2013). This local network in the cerebellar cortex has been postulated to generate oscillations between 10 and 30 Hz in the granule layer (De Zeeuw et al., 2008). Both of these circuits are capable of generating spindle-band oscillations, but further work is required to elucidate the underlying mechanisms.

## Cerebellar-neocortical sleep interactions during low frequency oscillations

**FIGURE 4:**
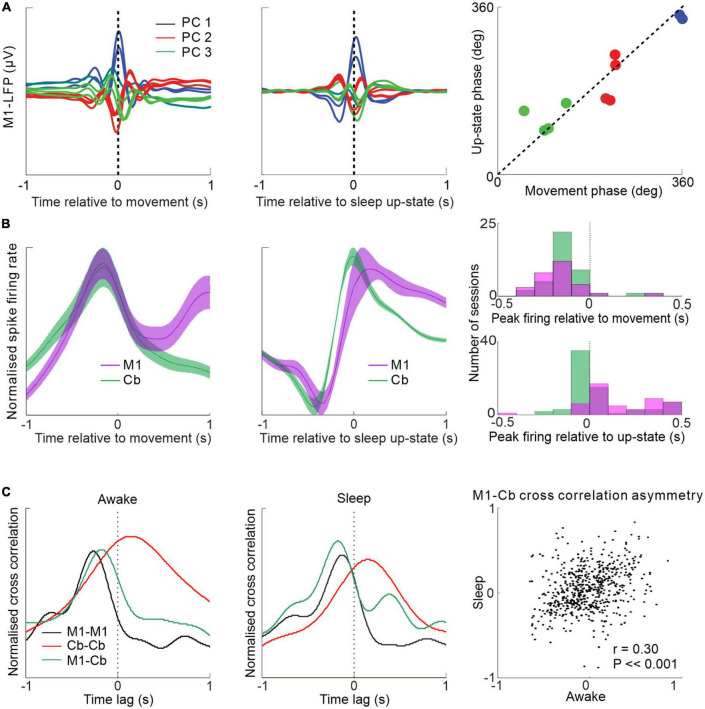
Low frequency M1-cerebellar interactions during movement and sleep. **(A)** Similar patterns of M1 LFPs aligned by waking movement and sleep slow oscillation. **(B)** M1 and cerebellar spike firing rates aligned to wrist movement during visuomotor task (left) and aligned to sleep up-state (middle) showing relative synchrony during the former and a relative cerebellar lag during the latter (right). **(C)** Spike cross-correlograms between pairs of neurons within M1, within cerebellum and between M1 and cerebellum during waking and sleep are similar (left and middle). M1-cerebellar cross-correlation temporal patterns are correlated between waking and sleep (right). Figures adapted with permission from Hall et al. (2014) and Xu et al. (2022).

It has previously been shown that low frequency M1 neuronal dynamics during movements are similar to those during low frequency oscillations in sleep, i.e., in the delta and low range (<4 Hz) (Hall et al., 2014; Figure 4A). A recent study examined the low frequency relationships between M1 and cerebellum during both sleep up-states and waking movements (Xu et al., 2022). The study showed that low-frequency oscillations in cerebellar spike firing during sleep up-states tend to lag that of M1 firing but the two areas are largely synchronous at low frequencies during waking movement (Figure 4B). This cerebellar delay during sleep may be explained by the altered state of the thalamus, reducing the efficacy of transmission at low-frequencies from the cerebellum to neocortex (but allowing sleep spindle transmission—see Figure 3). An alternative possibility is reduced transmission through the deep cerebellar nuclei (DCN)—supported by the fact that despite cerebellar Purkinje cells being inhibitory to DCN cells (Ito et al., 1970), Purkinje cells and DCN neurons both fire slowest during slow wave sleep (Hobson and McCarley, 1972; Palmer, 1979); implying that a sleep-related mechanism may act to lower transmission through the DCN. Interestingly, despite the altered M1-cerebellar relative spike timing between sleep up-states and movements the temporal relationships between pairs of M1-cerebellar neurons are nevertheless correlated between the two states (Figure 4C)—suggesting that waking neuronal dynamics may be recapitulated during sleep.

## The role of internal models in sleep-dependent learning

If the cerebellum is active in sleep, and is receiving inputs from the motor cortex that resemble those seen during movement, this raises the possibility that the sleeping cerebellum is performing some analogous computation to that which it performs in the awake state.

In this section we offer some tentative suggestions as to what this computation may be and how it could contribute to off-line learning. Influential theories of cerebellar function confer responsibility for learning internal models of the body and its interactions with the environment. Broadly these can be categorised as forward models that map causes (movements) to consequences (sensory predictions), or inverse models that map consequences (desired states) to causes (actions) (Wolpert and Kawato, 1998). During behaviour, internal models are thought to facilitate accurate feedforward control in the presence of delayed, noisy sensory feedback, either by generating motor corrections directly [e.g., feedback error learning (Kawato et al., 1987)] or predicting errors that can be rapidly corrected by a feedback controller [e.g., optimal feedback control (Todorov and Jordan, 2002)]. Thus, during movement, the motor cortex and the cerebellum engage in continuous bidirectional communication to fine-tune ongoing movement. At the same time, cerebellar models rapidly adapt based on experience, potentially via climbing fibre error signals. In sleep, however, the output of cerebellar-computed internal models seems instead to be relayed via sleep spindles, a frequency known to be effective at driving plasticity in neocortical circuits (Andrillon et al., 2011). It is therefore tempting to speculate whether information relayed from the cerebellum is driving lasting changes in the neocortex in sleep, similar to has been proposed for the consolidation episodic memories from short-term storage in the hippocampus to long-term storage in the neocortex.

**FIGURE 5:**
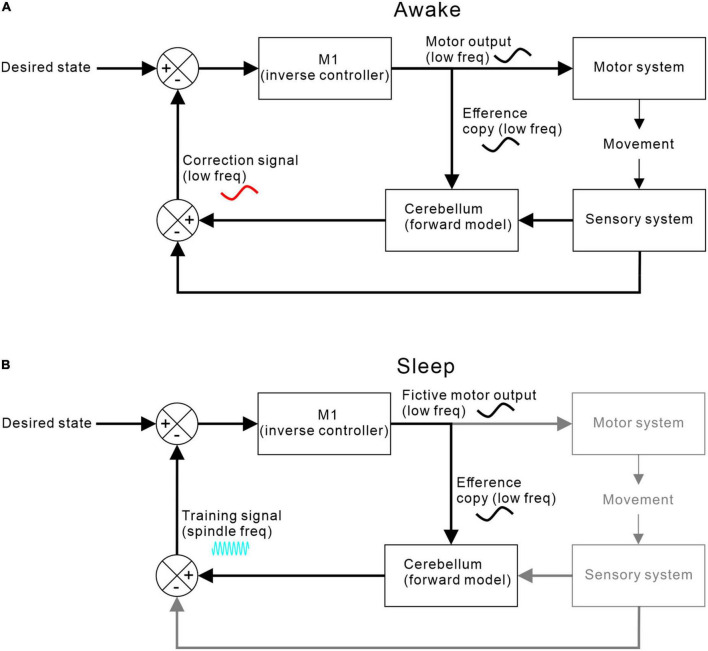
M1-cerebellar functional coupling. **(A)** Coupling during waking when M1 efference copy of motor commands and cerebellar correction signals are both at movement-related low frequencies. **(B)** Coupling during sleep when no movements or sensory feedbacks occur but low frequency fictive motor commands continue to be sent to the cerebellum, which then returns a training signal at spindle-band frequencies.

Figure 5 shows one possible arrangement for this, based around a forward model architecture (for review of forward models in motor control, see Miall and Wolpert (1996)]. During movement, an efference copy of the motor command generated by motor cortex is sent to a cerebellar forward model that predicts the expected consequence of the action. Discrepancies between predictions and goals drive motor corrections via a feedback loop. At the same time, discrepancies between predictions and actual consequences drive adaptation of the forward model.

Recent evidence suggests that cerebellar forward models may continue to generate sensory predictions in sleep. Dooley et al. (2021) studied neuronal activity in the ventro-lateral thalamus during sleep in young rats. Through early development, activity became synchronised with spontaneous limb movements, consistent with a predictive component to the reafferent signal. This predictive behaviour could be disrupted by blocking cerebellar output, thereby unmasking the delayed sensory response.

In adults, neocortical activity in sleep rarely causes overt movement and resultant sensory feedback. However, we hypothesise that fictive goal-directed motor commands (that resemble those seen during waking movement) can nevertheless be processed by cerebellar forward models to generate predicted sensory consequences. The discrepancy between fictive goals and predicted consequences could act as a simulated reward signal (relayed via sleep spindles) to drive off-line optimisation of the controller in sleep. As a result, daytime learning stored in cerebellar forward models could during sleep be transferred and transformed into improved control policies (learned patterns of motor output) represented in the neocortex.

The concept of using a previously learnt predictive model to optimise control policies is long-standing in the computational literature. Forward mappings (i.e., cause-effect relationships) are in general well-posed and, crucially, sensory predictions can be compared directly to actual consequences. As a result, forward models can be learned through efficient error-based mechanisms wherein sensory feedback from the world acts as a teaching signal. By contrast, inverse control policies (goal-action relationships) are often ill-posed (there may be many ways to achieve a desired goal, or none at all) and the world provides only an indirect training signal (an unsuccessful outcome does not directly tell you what action you should have taken instead). The distal supervised learning architecture of Jordan and Rumelhart (1992) places an inverse controller in series with a forward model and back-propagates errors through the composite system until the predicted outcome of the control policy resembles sensory goals that are “envisioned” by the learner. This shares similarities with the Dyna architecture of Sutton (1991) in which reinforcement learning is applied to simulated experience generated by a forward model that predicts the consequences of hypothetical actions. While originally considered as models of planning or mental practice while awake, these ideas have been influential in shaping theories of hippocampal replay in sleep (Johnson and Redish, 2005; Russek et al., 2017) but only rarely been applied to the cerebellum (Passot et al., 2013). We believe they could be a framework for interpreting data on cerebellar activity and cerebro-cerebellar communication in sleep and generate testable hypotheses for its influence on daytime behaviour.

This scheme could also be extended to learning that does not depend on the neocortex, such as eye-blink conditioning (Hesslow and Ivarsson, 1996). Evidence suggests the cerebellar cortex is required for the acquisition of this behaviour (Attwell et al., 2001), but the conditioned response is retained to some extent after cerebellar cortical lesion (Perrett et al., 1993), consistent with transfer of the memory engramme to the deep cerebellar nuclei. One possibility is that the cerebellar cortex learns a forward predictive model of the sensory consequences of the conditioned stimulus (usually an auditory tone). Such a predictive model could contribute to training an inverse controller (computed by the direct pathway through the deep cerebellar nuclei) that maps sensory information to the appropriate motor command to blink. This process could continue in sleep even in the absence of real sensory consequences, and such a framework may explain why the consolidation of eye-blink conditioning appears to be sleep-dependent (De Zeeuw and Canto, 2020).

## Conclusion and future directions

The cerebellum seems to be an active participant in the sleep process and is emerging as an important player in sleep-dependent memory formation. While confirmed by recent experiments, it appears that disjointed evidence for a cerebellar contribution to sleep processes has been present for decades. With this in mind future targetted experiments will hopefully shed more light on this contribution. While cerebellar lesions can disrupt sleep architecture (see above), it is unclear whether cerebro-cerebellar signals, for example at sleep spindle frequencies, influence the time spent in different sleep stages, for example stage 2 sleep. Closed-loop perturbations are increasingly used to explore the role of hippocampal ripples (Aleman-Zapata et al., 2022), and a similar approach, for example perturbing cerebellar circuity during sleep spindles, might strengthen the case for a causal role for cerebellar outputs. Similar experiments may be able to address whether selectively blocking cerebellar sleep spindles affect off-line improvements in procedural learning.

Our hypothesis that the cerebellum acts as an off-line “simulator” enabling off-line practice of motor control policies begs speculation about a role in dreams. However, some caution is warranted here since the strongest evidence for a role of sleep in cerebellar-dependent procedural learning relates to stage 2 sleep. While it is true that dreams can take place in stage 2 sleep (Morel et al., 1991; Blagrove et al., 2011), most occur in REM sleep (Crick and Mitchison, 1983). Moreover, recent research has implicated dreaming in consolidation of cognitive (Ackermann and Rasch, 2014) and in particular emotional memories (Tempesta et al., 2018). Nevertheless, as previously discussed, the cerebellum is certainly active during REM sleep. Moreover, the cerebellum has been implicated in modulating both cognition and affect (Schmahmann, 2004), and these functions are likely supported by extensive connections with non-motor regions of the brain including the prefrontal cortex (Klein et al., 2016). Indeed, the architecture of the cerebellar-thalamo-cortical loop is recapitulated across these non-motor areas (Strick et al., 2009), and it would be interesting to explore whether the cerebellum might exert a wider influence on off-line sleep processes through the implementation of forward predictive models.

Indeed, there are many disorders for which cerebellar damage, sleep disorders (including spindle abnormalities) and impaired off-line consolidation are overlapping, from autism to schizophrenia (Andreasen and Pierson, 2008; Wang et al., 2014; Farmer et al., 2018; Manoach and Stickgold, 2019). The non-motor functions of the cerebellum have traditionally been overlooked in part because the most prominent deficits following adult cerebellar injury affect movement. Nevertheless, Wang et al. (2014) introduced the concept of “developmental diaschisis” to describe how cerebellar disruption in early life might lead to impaired development of other, remote cortical areas. Given the importance of sleep and sleep spindles for the developing brain, a role in sleep-dependent consolidation could provide a key to unlocking in future the contribution of the cerebellum to these non-motor functions.

## Author contributions

Both authors listed have made a substantial, direct, and intellectual contribution to the work, and approved it for publication.
